# Supplementary material for: DNA Methylation of PXDN Is Associated with Early-Life Adversity in Adult Mental Disorders
Source: Biomolecules. 2024 Aug 9;14(8):976. doi: 10.3390/biom14080976 (PMC11353138; doi:10.3390/biom14080976)
Supplement: Supplementary file 1 [file biomolecules-14-00976-s001.zip › 24-7-29.pdf]

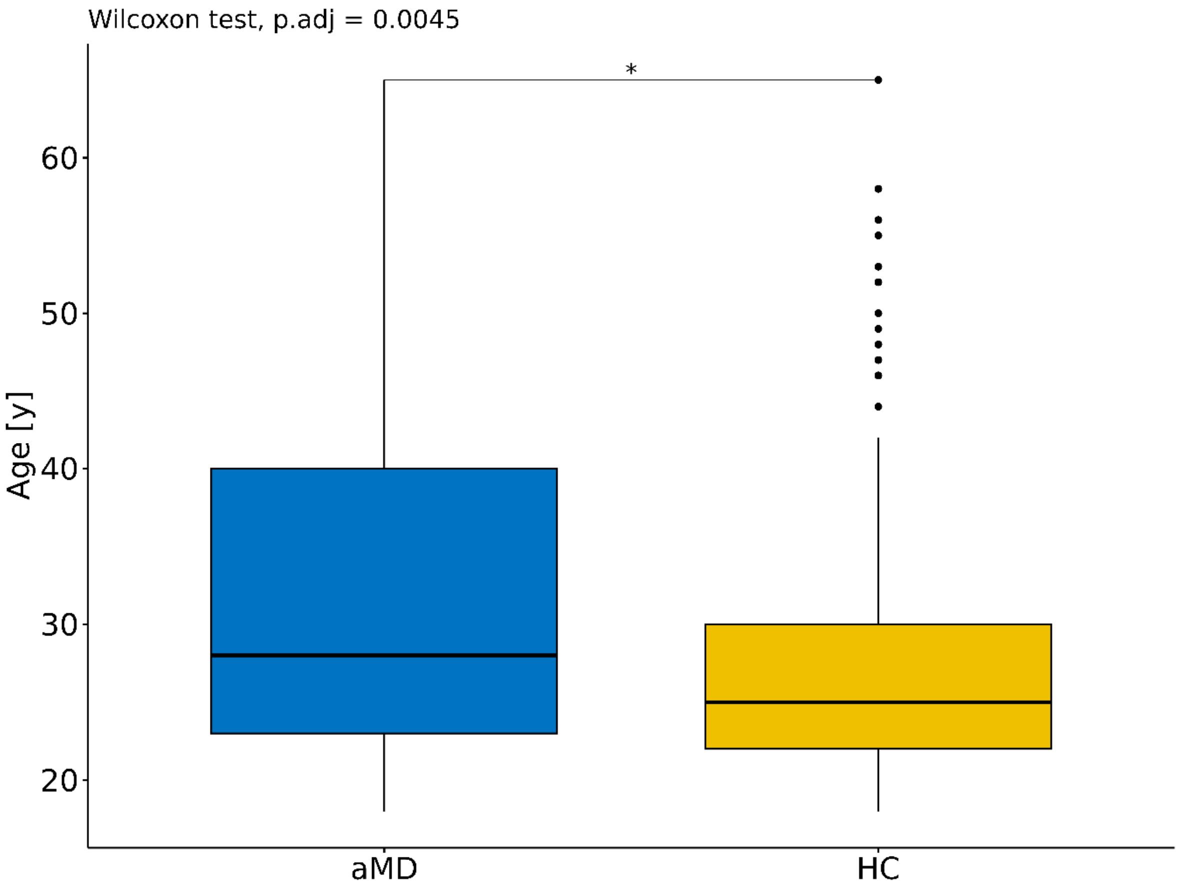

Figure S1: Age distribution between aMD and HC. Wilcoxon rank sum tests and Benjamini-Hochberg correction (n_{tests} = 4) were applied. *p.adj. <0.01.

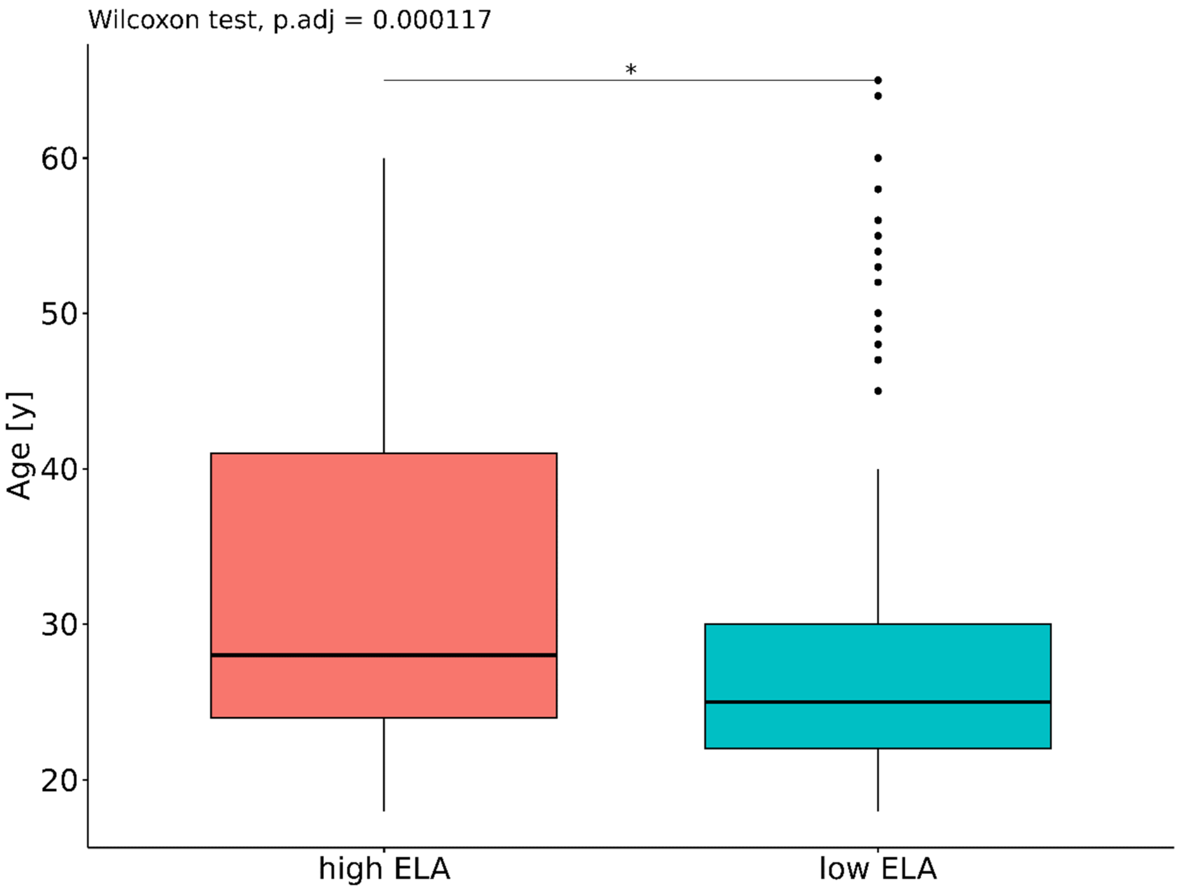

Figure S2: Age distribution in the groups with high and low levels of ELA. Wilcoxon rank sum tests and Benjamini-Hochberg (n_{tests} = 4) correction were applied. *p.adj. <0.001.

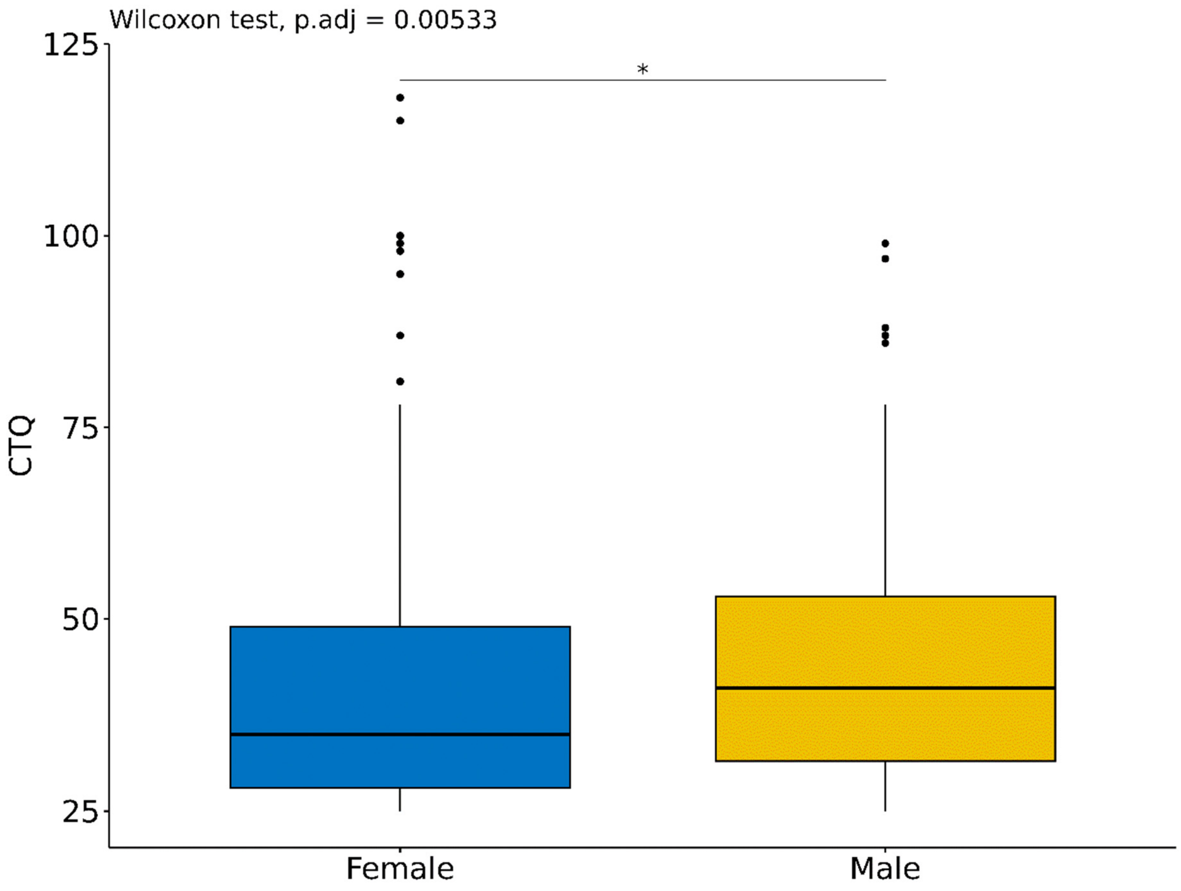

Figure S3: Total CTQ scores of male and female participants of the overall cohort. Wilcoxon rank sum tests and Benjamini-Hochberg correction (n_{tests} = 4) were applied. *p.adj. <0.01.

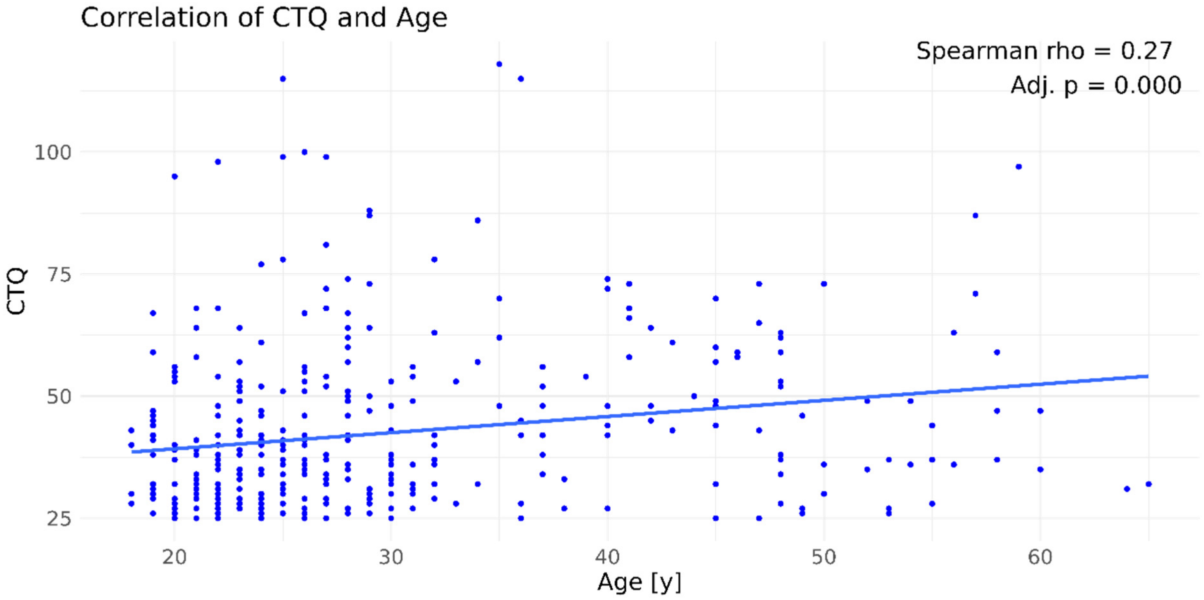

Figure S4: Spearman correlation of total CTQ and age in the overall cohort.

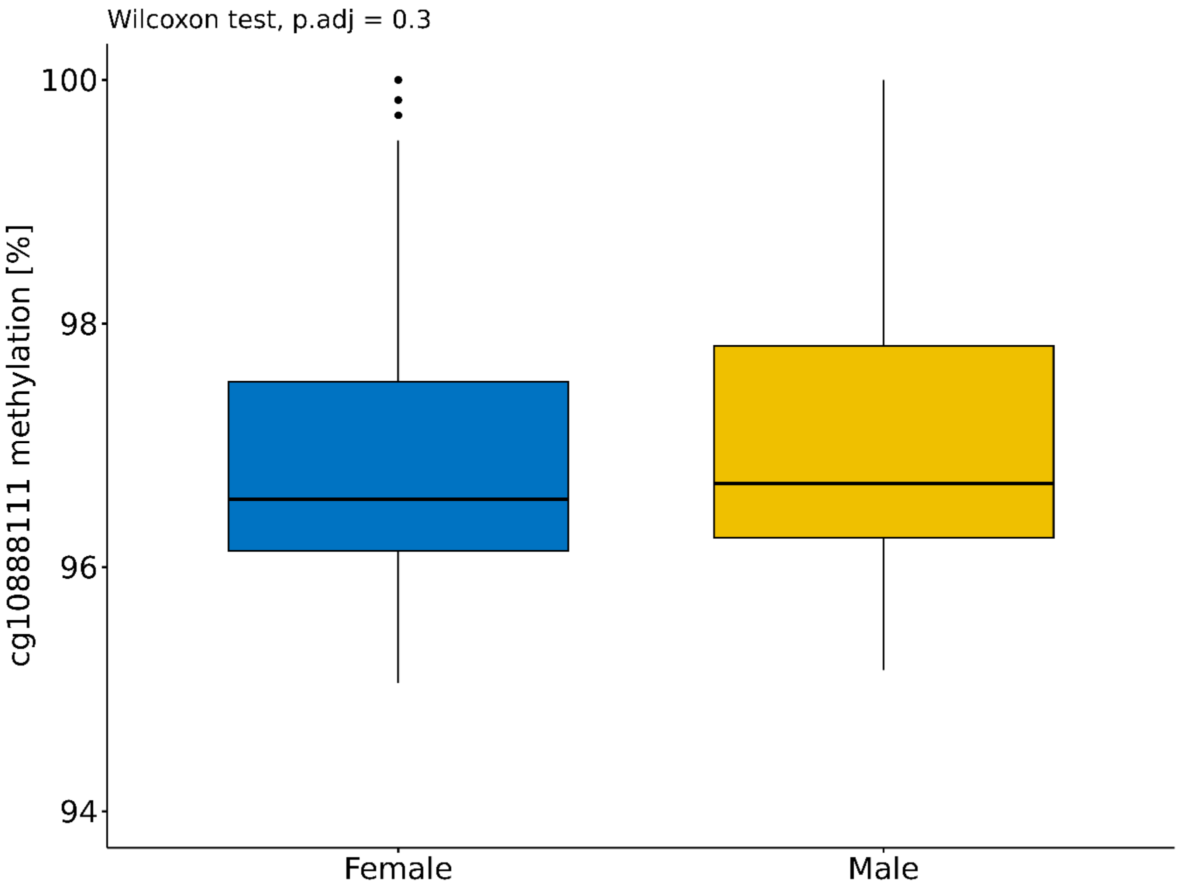

Figure S5: Cg10888111 methylation levels in the context of sex in the overall cohort. Wilcoxon rank sum tests and Benjamini-Hochberg correction (n_{tests} = 4) were applied.

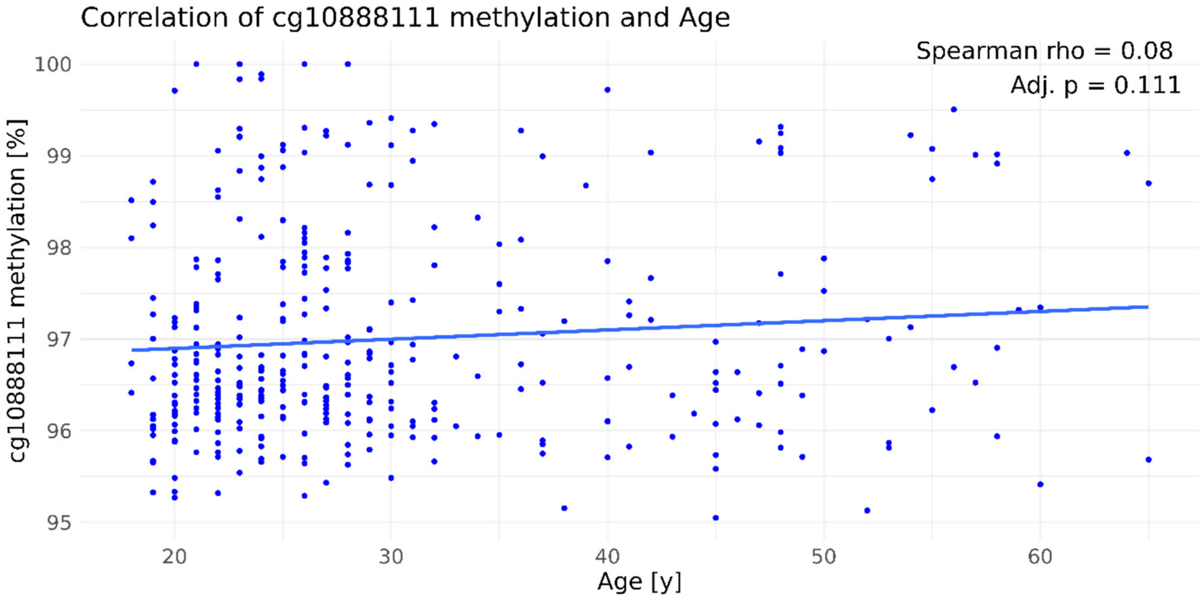

Figure S6: Spearman correlation of cg10888111 methylation levels and age in the overall cohort.

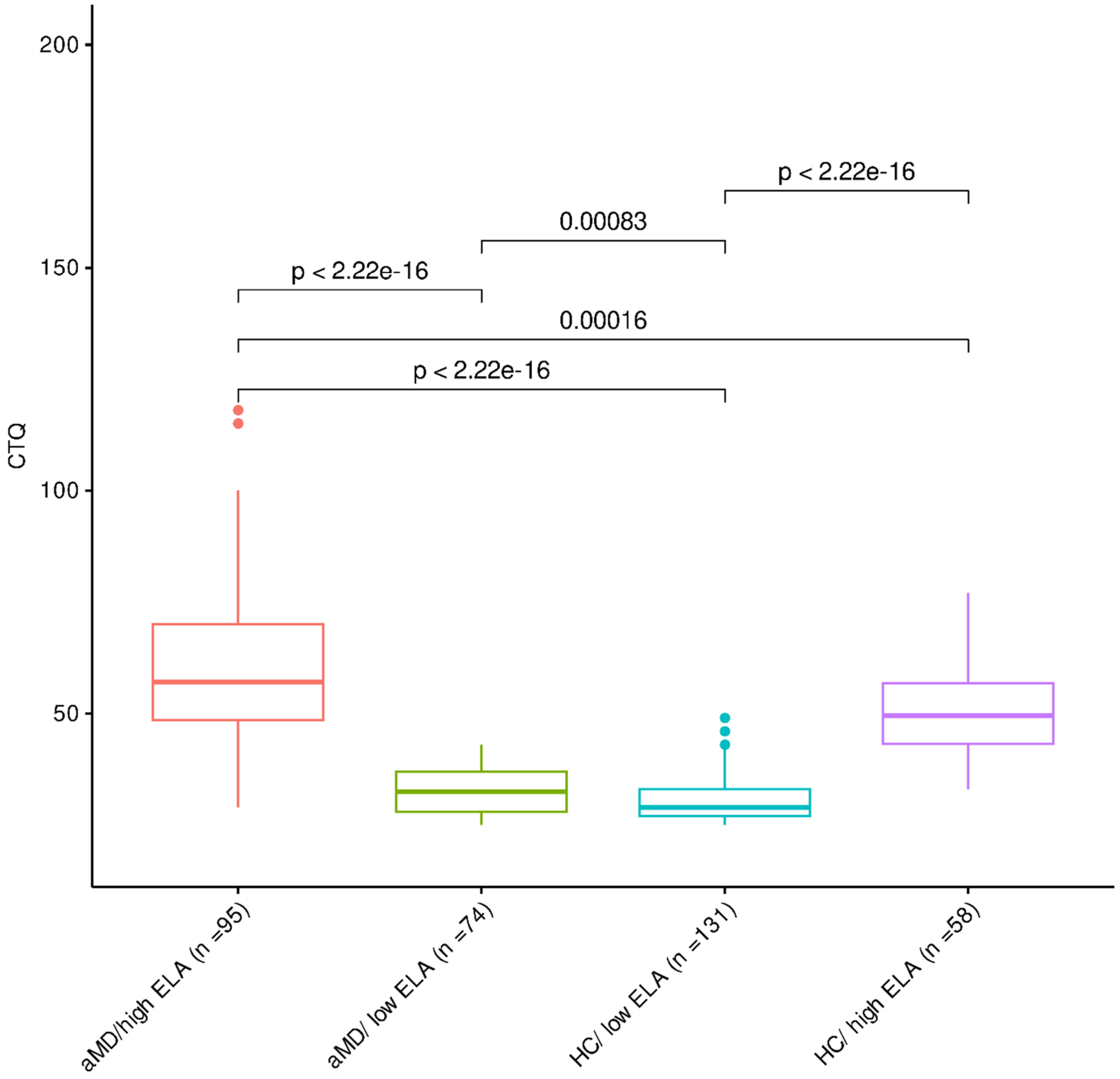

Figure S7: Total CTQ compared between the four groups aMD/ high ELA, aMD/ low ELA, HC/ high ELA and HC/ low ELA. Wilcoxon rank sum test was applied in a pairwise comparison. Benjamini-Hochberg procedure was applied to correct for multiple testing.

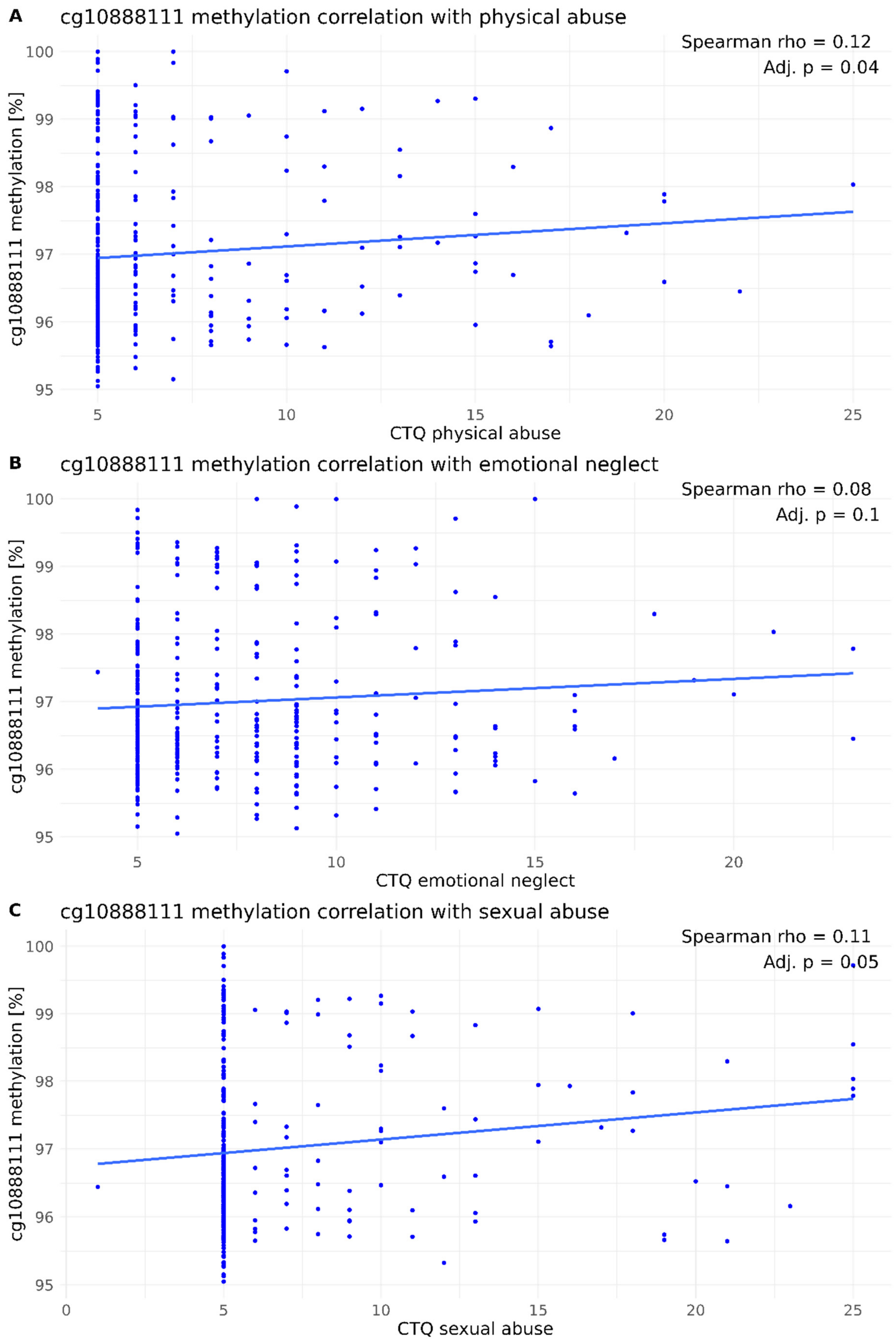

Figure S8: Spearman correlation of cg10888111 methylation levels with CTQ scores from the subclasses **A.** physical abuse, **B.** physical neglect and **C.** sexual abuse.

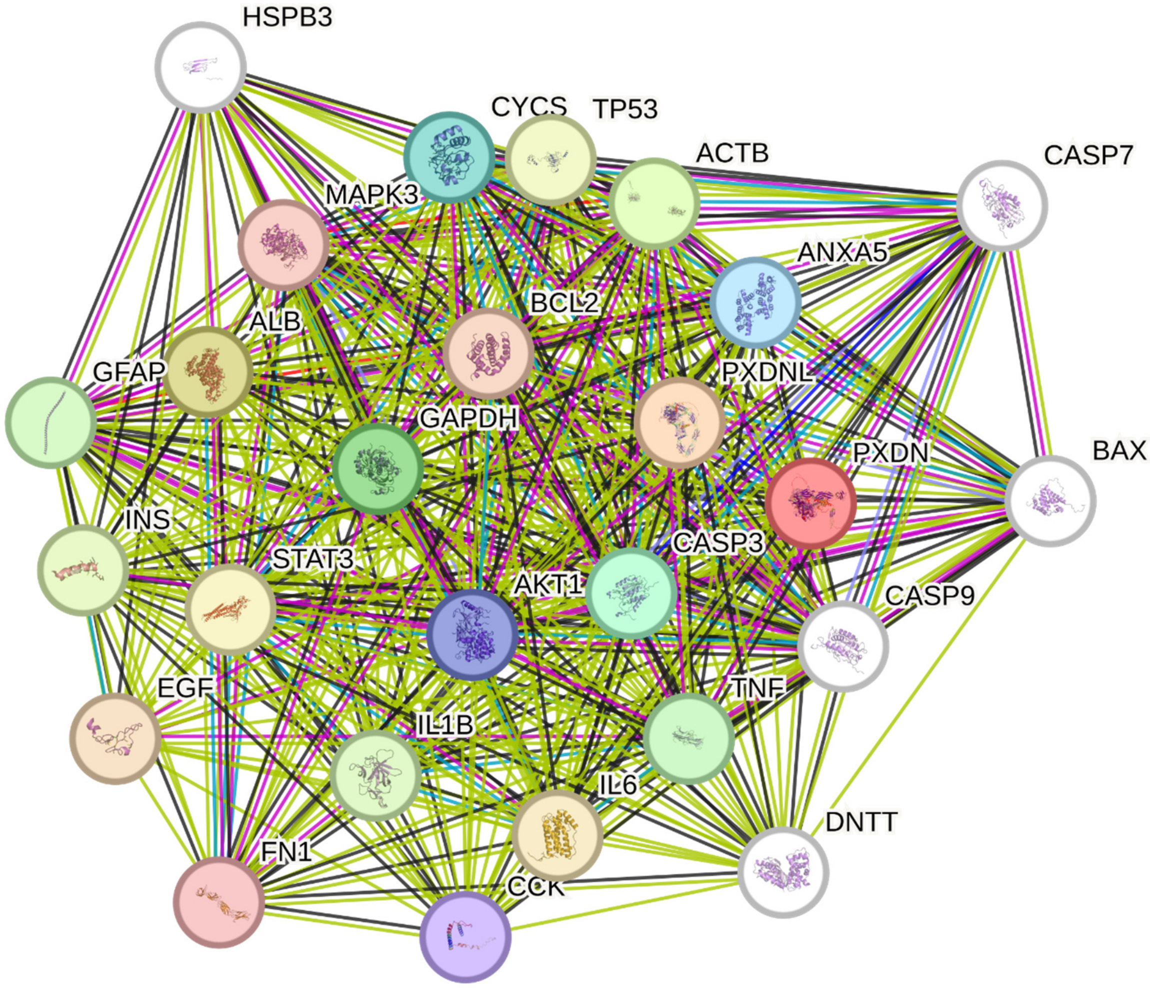

Figure S9: STRING network of PXDN and its interaction partners generated with a medium confidence of 0.4 and 20 interaction partners shown for the 1st layer as well as five for the second 2nd layer.
